# PROTAC-DB: an online database of PROTACs

**DOI:** 10.1093/nar/gkaa807

**Published:** 2020-10-03

**Authors:** Gaoqi Weng, Chao Shen, Dongsheng Cao, Junbo Gao, Xiaowu Dong, Qiaojun He, Bo Yang, Dan Li, Jian Wu, Tingjun Hou

**Affiliations:** Innovation Institute for Artificial Intelligence in Medicine of Zhejiang University, College of Pharmaceutical Sciences, Zhejiang University, Hangzhou 310058, Zhejiang, China; State Key Lab of CAD&CG, Zhejiang University, Hangzhou 310058, Zhejiang, China; Innovation Institute for Artificial Intelligence in Medicine of Zhejiang University, College of Pharmaceutical Sciences, Zhejiang University, Hangzhou 310058, Zhejiang, China; Xiangya School of Pharmaceutical Sciences, Central South University, Changsha 410004, Hunan, China; Innovation Institute for Artificial Intelligence in Medicine of Zhejiang University, College of Pharmaceutical Sciences, Zhejiang University, Hangzhou 310058, Zhejiang, China; Innovation Institute for Artificial Intelligence in Medicine of Zhejiang University, College of Pharmaceutical Sciences, Zhejiang University, Hangzhou 310058, Zhejiang, China; Innovation Institute for Artificial Intelligence in Medicine of Zhejiang University, College of Pharmaceutical Sciences, Zhejiang University, Hangzhou 310058, Zhejiang, China; Innovation Institute for Artificial Intelligence in Medicine of Zhejiang University, College of Pharmaceutical Sciences, Zhejiang University, Hangzhou 310058, Zhejiang, China; Innovation Institute for Artificial Intelligence in Medicine of Zhejiang University, College of Pharmaceutical Sciences, Zhejiang University, Hangzhou 310058, Zhejiang, China; College of Computer Science and Technology, Zhejiang University, Hangzhou 310058, Zhejiang, China; Innovation Institute for Artificial Intelligence in Medicine of Zhejiang University, College of Pharmaceutical Sciences, Zhejiang University, Hangzhou 310058, Zhejiang, China; State Key Lab of CAD&CG, Zhejiang University, Hangzhou 310058, Zhejiang, China

## Abstract

Proteolysis-targeting chimeras (PROTACs), which selectively degrade targeted proteins by the ubiquitin-proteasome system, have emerged as a novel therapeutic technology with potential advantages over traditional inhibition strategies. In the past few years, this technology has achieved substantial progress and two PROTACs have been advanced into phase I clinical trials. However, this technology is still maturing and the design of PROTACs remains a great challenge. In order to promote the rational design of PROTACs, we present PROTAC-DB, a web-based open-access database that integrates structural information and experimental data of PROTACs. Currently, PROTAC-DB consists of 1662 PROTACs, 202 warheads (small molecules that target the proteins of interest), 65 E3 ligands (small molecules capable of recruiting E3 ligases) and 806 linkers, as well as their chemical structures, biological activities, and physicochemical properties. Except the biological activities of warheads and E3 ligands, PROTAC-DB also provides the degradation capacities, binding affinities and cellular activities for PROTACs. PROTAC-DB can be queried with two general searching approaches: text-based (target name, compound name or ID) and structure-based. In addition, for the convenience of users, a filtering tool for the searching results based on the physicochemical properties of compounds is also offered. PROTAC-DB is freely accessible at http://cadd.zju.edu.cn/protacdb/.

## Graphical Abstract

**Graphical Abstract:**
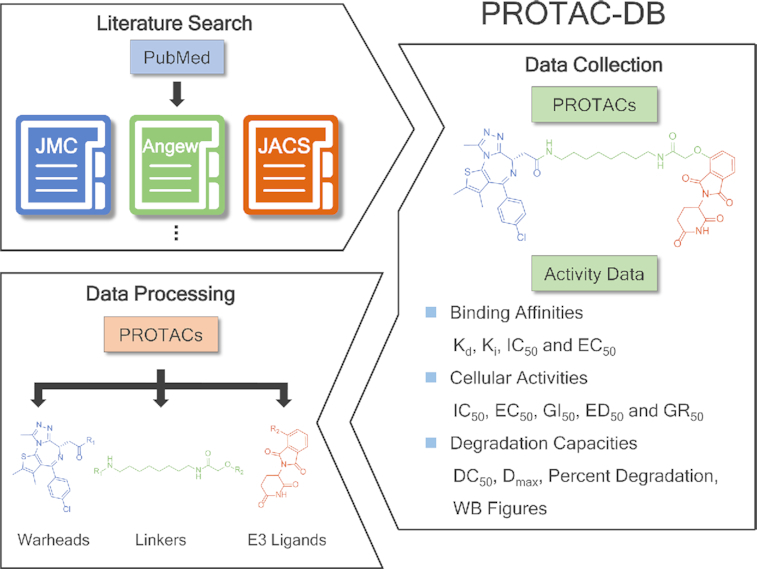
The three basic steps to construct PROTAC-DB: (I) literature searching, (II) data collection, and (III) data processing by separating PROTACs into warheads, E3 ligands and linkers.

## INTRODUCTION

In the past few years, proteolysis-targeting chimeras (PROTACs), which selectively induce targeted protein degradation through the ubiquitin-proteasome system, represent a new drug discovery strategy and have attracted extensive attention from medicinal chemists and pharmaceutical industry (1–4). PROTACs are heterobifunctional molecules, which contain a small molecule targeting the protein of interest (warhead), a small molecule capable of recruiting an E3 ligase (E3 ligand), and a linker connecting the above two moieties. In contrast to traditional occupancy-based inhibitors that have sufficient binding affinities to druggable active sites, PROTACs require only transient binding to target proteins to catalytically induce ubiquitination and degradation (5,6). Furthermore, since it is unnecessary for warheads to occupy druggable binding sites which modulate protein functions, PROTACs can exploit all surface binding sites on the targeted proteins and hence have the potential to modulate ‘undruggable’ targets (6).

The first PROTAC was reported by Sakamoto *et al.* in 2001, and it consists of a covalent inhibitor of methionine aminopeptidase 2 (MetAP2) and a ten-residue phosphopeptide fragment capable of recruiting an F-box protein β-transducin repeat-containing protein (β-TRCP) (1). However, due to the limitations of peptide-based PROTACs *in vivo*, researchers have increasingly focused on the development of potent small-molecule PROTACs. In 2008, Schneekloth *et al.* reported the first small-molecule PROTAC, which degraded androgen receptor (AR) through its recruitment to the E3 ligase, MDM2 (2). Shortly thereafter, inhibitors of apoptosis protein (IAP)-based PROTACs termed SNIPERs (specific and nongenetic IAP-dependent protein erasers) have also been developed (4). As the rapid development of the PROTACs technology, nowadays, a number of PROTACs achieved potent and highly selective degradation of targeted proteins in cellular assays and even *in vivo* (5,7–13). Encouragingly, two PROTACs named ARV-110 and ARV-471, targeting AR and estrogen receptor (ER), respectively, have been advanced into phase I clinical trials (14).

Despite the tremendous progress made over the past decade, designing PROTACs with desirable physicochemical, absorption, distribution, metabolism, and excretion properties still remains a big challenge. Different from traditional small molecule drugs, PROTACs may not conform to the Lipinski's ‘rule of five’ due to their high molecular weight, which would limit their cellular permeability and other drug-like properties (15,16). Although the optimization of PROTACs should focus more on the whole molecule rather than the individual components, it is useful to consider them individually in preliminary PROTAC design (17). In this regard, the linker design is considered as the foremost critical domain for the design of PROTACs (15). Moreover, accumulated evidences illustrate that linkers are associated with the entropy, selectivity, activity, aqueous solubility, permeability of PROTACs, and so on (17,18). However, the exploration of the linker designs is endless. In order to boost the rational design of PROTACs, it is quite essential to collect and annotate experimental data and structural information about PROTACs.

Although there are some comprehensive databases that also collect some PROTACs information, such as GtoPdb (19), PubChem (20) and ChEMBL (21), the quantity and experimental information of PROTACs in these databases are still quite limited. Here, a newly developed database, PROTAC-DB, is therefore introduced with a user-friendly web interface. To the best of our knowledge, this is the first online database that collects the diverse information related to PROTACs, including their chemical structures, biological activities, and physicochemical properties. Furthermore, to better facilitate user analysis, the structures of PROTACs are divided into three domains, including warheads, E3 ligands and linkers. The PROTACs with the same warheads, E3 ligands or linkers can be categorized into different detailed information pages, which can also be served as a valuable resource for the rational design of PROTACs. Moreover, all data of PROTACs, warheads, E3 ligands and linkers are available for downloading as either SDF or CSV files.

## MATERIALS AND METHODS

### Data collection and processing

**Figure 1.**
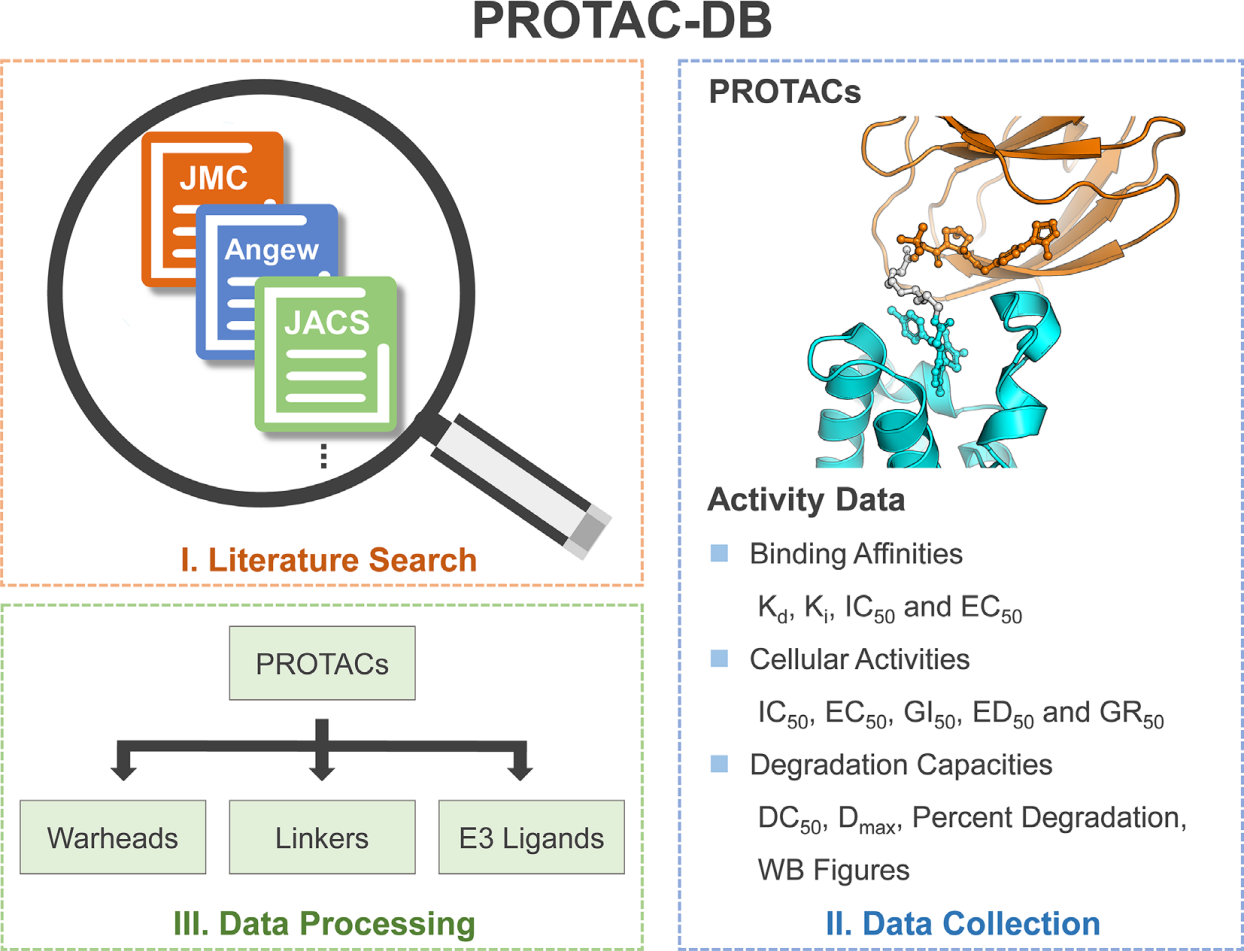
The basic data collection and processing stages of PROTAC-DB: (I) the literature searching stage, (II) the data collection stage and (III) the data processing stage by separating PROTACs into warheads, E3 ligands and linkers.

The basic data collection and processing stages of PROTAC-DB are illustrated in Figure 1. The information of PROTACs was searched in PubMed using the keywords of ‘degrader* OR protac OR proteolysis targeting chimera’. The literature about small-molecule PROTACs was collected and that about peptide-based and HaloTag PROTACs was ruled out. Subsequently, the chemical structures and biological activities of PROTACs were manually extracted from the literature. The biological activities here contain the degradation capacities, binding affinities, and cellular activities. The detailed information is as follows.

Degradation capacity: In general, DC_50_ (concentration that results in a 50% targeted protein degradation) and D_max_ (the maximal level of protein degradation) are utilized to quantify the power of targeted protein degradation of PROTACs. However, since a large number of PROTACs lacked the above data, the percentage degradation was also integrated into the database if it was assessed at least at two concentrations and at least two independent experiments were conducted for each concentration. Furthermore, the Western blotting (WB) figures were also collected to show the degradation capacities of PROTACs. But it should be noted that the WB figures are only displayed on the detailed information pages of PROTACs, not on the searching result pages.

Binding affinity: The binding affinities between PROTACs and targeted proteins, PROTACs and E3 ligases, and the formation of ternary complexes were collected. The binding affinity of the formation of ternary complex can be employed to assess the capacity of PROTAC-induced complex formation with E3 ligase and targeted protein. It can be determined through some assays between E3 ligase (targeted protein) and the complex of PROTAC and targeted protein (E3 ligase) (22,23). There are four types of values, including *K*_d_, *K*_i_, IC_50_ and EC_50_. Only *K*_d_ and IC_50_ are displayed on the searching result pages and the other are shown on the detailed information pages. In addition, the biophysical binding data, including Δ*G*, Δ*H*, –*T*Δ*S*, *t*_1/2_, *k*_on_ and *k*_off_, were also collected into the database and displayed on the detailed information page.

Cellular activity: Five types of values, including IC_50_, EC_50_, GI_50_, ED_50_ and GR_50_, were collected. Similarly, ED_50_ and GR_50_ are only displayed on the detailed information pages, not on the searching result pages.

Additionally, ten important physicochemical properties related to drug-likeness calculated by using the RDKit toolkit (http://www.rdkit.org) and ALOGPS (24) were provided on the detailed information pages, including molecular weight, exact mass, partition coefficient (log *P*), aqueous solubility (logS), heavy atom count, ring count, hydrogen bond acceptor count, hydrogen bond donor count, rotatable bond count and topological polar surface area. Furthermore, the PDB codes of the ternary crystal structures of PROTACs were also incorporated to the database.

Based on the collected information of PROTACs, the structures of PROTACs were further separated into warheads, E3 ligands and linkers according to the literature and the initial structures of warheads and E3 ligands (structures before being modified and integrated into PROTACs). The biological activities of the initial structures of warheads and E3 ligands were collected from the literature and other databases, such as PubChem (20), ChEMBL (21) and BindingDB (25). The important physicochemical properties of linkers and the initial structures of warheads and E3 ligands were also calculated in the same way. All in all, 1662 PROTACs, 202 warheads, 65 E3 ligands and 806 linkers were collected into PROTAC-DB.

### Development of PROTAC-DB

PROTAC-DB was built using the Python web framework of Tornado (an asynchronous networking library, https://www.tornadoweb.org/en/stable/) and deployed on a Linux server, accessible at http://cadd.zju.edu.cn/protacdb/. All the data was stored in PostgreSQL (an object-relational database, https://www.postgresql.org/). For the visualization of 2D chemical structures, the OpenEye Python toolkits were employed to generate the images of structures. Moreover, ChemDoodle was utilized as the molecule editor, which helps users to query the database with self-edited molecules (26).

## RESULTS

### Query and browse of database

In order to facilitate the retrieval of the data in PROTAC-DB, we provide the searching and browsing tools. As to the searching tools, PROTAC-DB can be queried with text-based and structure-based search. Text-based search serves as a simple way to search throughout PROTAC-DB by entering a single term, such as target name, compound name or ID. For structure-based search, users can input a SMILES string, upload a MOL/SDF file or sketch a molecule within the ChemDoodle editor. After the self-edited molecule has been imported, one of the three searching options (e.g. similarity, substructure or exact) can be chosen. In the similarity search, the bit vector Morgan fingerprint, an FCFP-like fingerprint, is utilized to compute the Tanimoto similarity between two molecules. A dataset (PROTACs, warheads, E3 ligands or linkers) can be selected for searching.

The browsing tools summarize the data in PROTAC-DB through two categories: ‘Target browse’ and ‘Compound browse’. The target browse will display the list of the names of the targeted proteins under the class tabs of ‘PROTACs’, ‘Warheads’, ‘E3 ligands’ and ‘Linkers’. Then, clicking on the selected proteins in the list will jump to the list of all compounds corresponding to the protein. The compound browse is mainly utilized to visualize the 2D structures of all compounds under the class tabs of ‘PROTACs’, ‘Warheads’, ‘E3 ligands’ and ‘Linkers’. In addition, under the class tabs of ‘PROTACs’, ‘Warheads’ and ‘E3 ligands’, the biological activities will also be displayed.

### Visualizing and filtering the results within a datasheet

**Figure 2.**
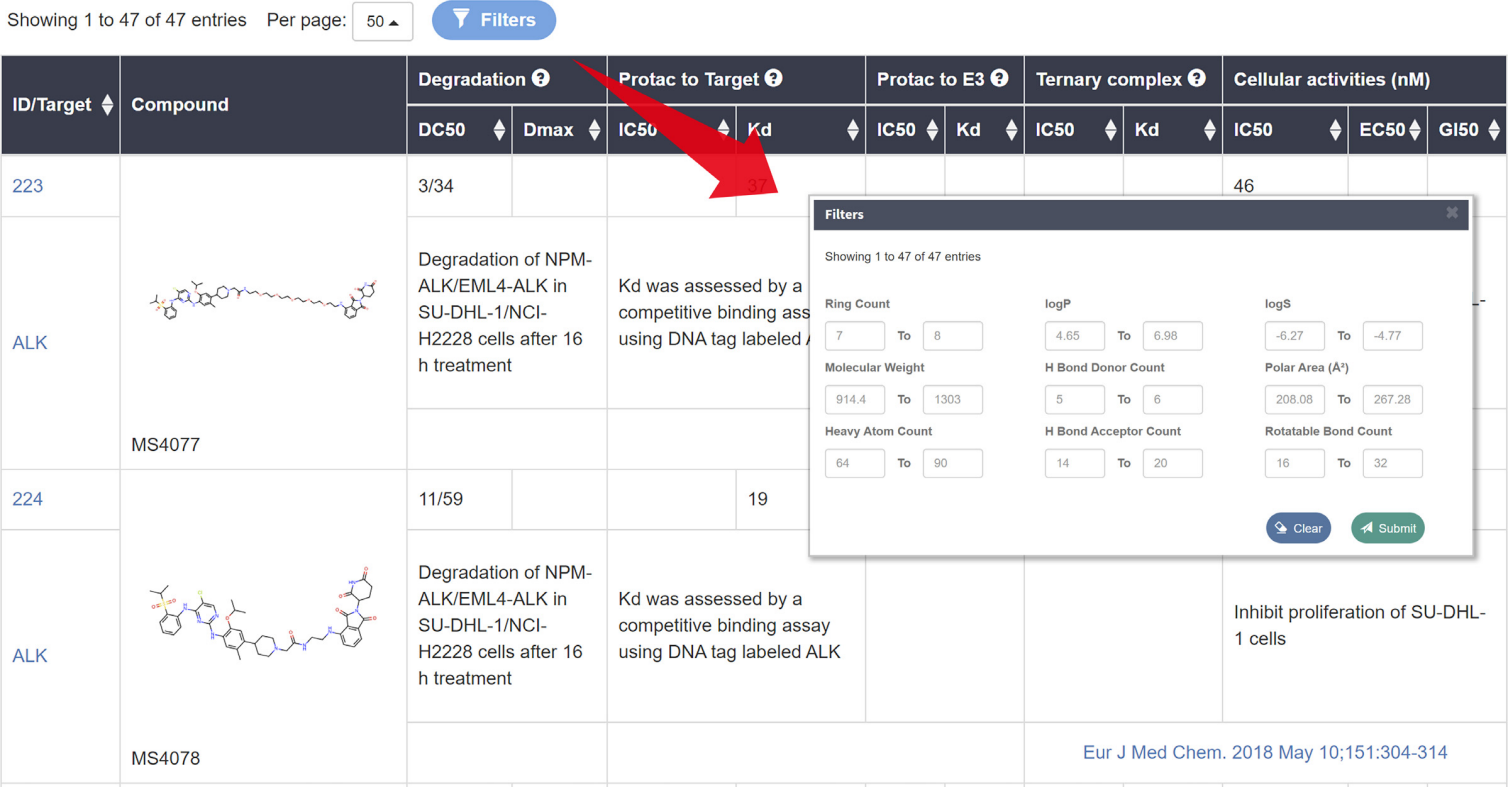
The query or browsing results and the filtering tool for the PROTAC dataset.

The query or browsing results are displayed as a datasheet that contains the 2D structures and other information, such as compound IDs, targeted proteins and biological activities (Figure 2). Clicking on the image of the structure can get an enlarged one. Besides, in order to help users to refine the search, the filtering tool based on the physicochemical properties (e.g. molecular weight, log *P*, log *S*, topological polar surface area) is provided by PROTAC-DB. The minimum and maximum values of each property in the searching results will also be displayed in the filtering tool.

For PROTACs, except the 2D structures, compound IDs and targeted proteins, the biological activities are also displayed in the datasheet, which contains the degradation capacities, binding affinities and cellular activities. The datasheet can be sorted according to the values of biological activities.

For warheads and E3 ligands, only the initial structures are shown in the searching results. The structures integrated into PROTACs after modification are summarized in their corresponding detailed information pages. Moreover, the biological activities of the initial structures are also displayed in the datasheet. Similarly, the datasheet can also be sorted according to these criteria.

For linkers, only the 2D structures, compound IDs and targeted proteins are shown in the datasheet. The ‘R1’ and ‘R2’ in the structures represent the sites that conjugate warheads and E3 ligands, respectively.

### Accessing all data of a compound

If users are interested in a compound, clicking on its compound ID in the result datasheet will jump to the detailed information pages where all data about this compound are summarized.

**Figure 3.**
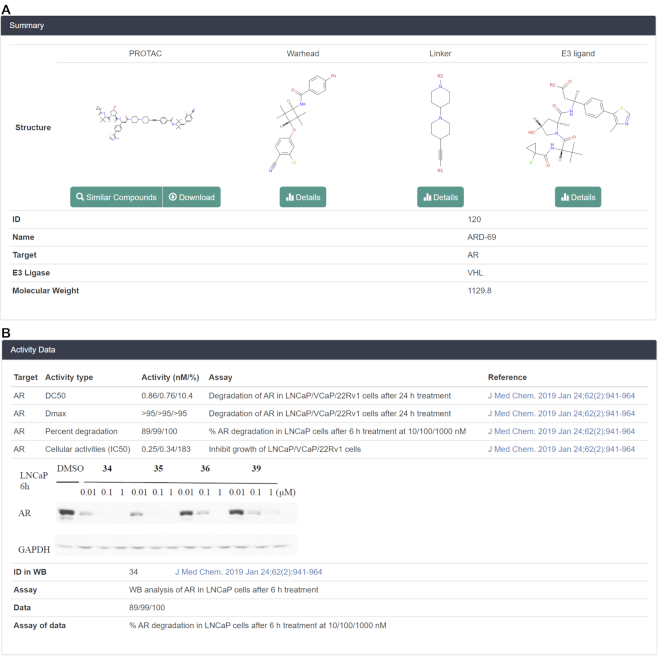
(**A**) Summary and (**B**) activity data tabs in the detailed information pages of PROTACs.

In the detailed information pages of PROTACs, four different tabs are incorporated, including summary, representation, calculated properties and activity data. As shown in Figure 3A, the summary tab displays the structures of PROTAC, warhead, linker and E3 ligand. The ‘R1’ and ‘R2’ in the warhead and E3 ligand represent the corresponding sites connecting to the linker.

Moreover, for the convenience of users, the similarity searching tool is also integrated into the summary tab to find the similar compounds of this PROTAC in the database. The similarity threshold here is ≥0.80. The MOL and SDF files of the compound are also available for downloading in the summary tab. Furthermore, if users are interested in the warhead, linker or E3 ligand, they can click the ‘Details’ button to open the corresponding detailed information page to get more information. Below the structures, the compound ID, name, targeted protein and E3 ligase used by the PROTAC and the molecular weight are summarized. If the PROTAC has a ternary crystal structure, the PDB codes will also be shown in the summary tab.

The representation tab contains the IUPAC name, InChI, InChI key, canonical SMILES and molecular formula. For the calculated properties tab, ten important physicochemical properties are displayed, including molecular weight, exact mass, log *P*, log *S*, heavy atom count, ring count, hydrogen bond acceptor, hydrogen bond donor count, rotatable bond count and topological polar surface area.

In the activity data tab (Figure 3B), all biological activities for the selected compound are shown, including the degradation capacities, binding affinities and cellular activities. Apart from the numerical activity data, the WB figure is also displayed to characterize the degradation capacities of the PROTAC.

**Figure 4.**
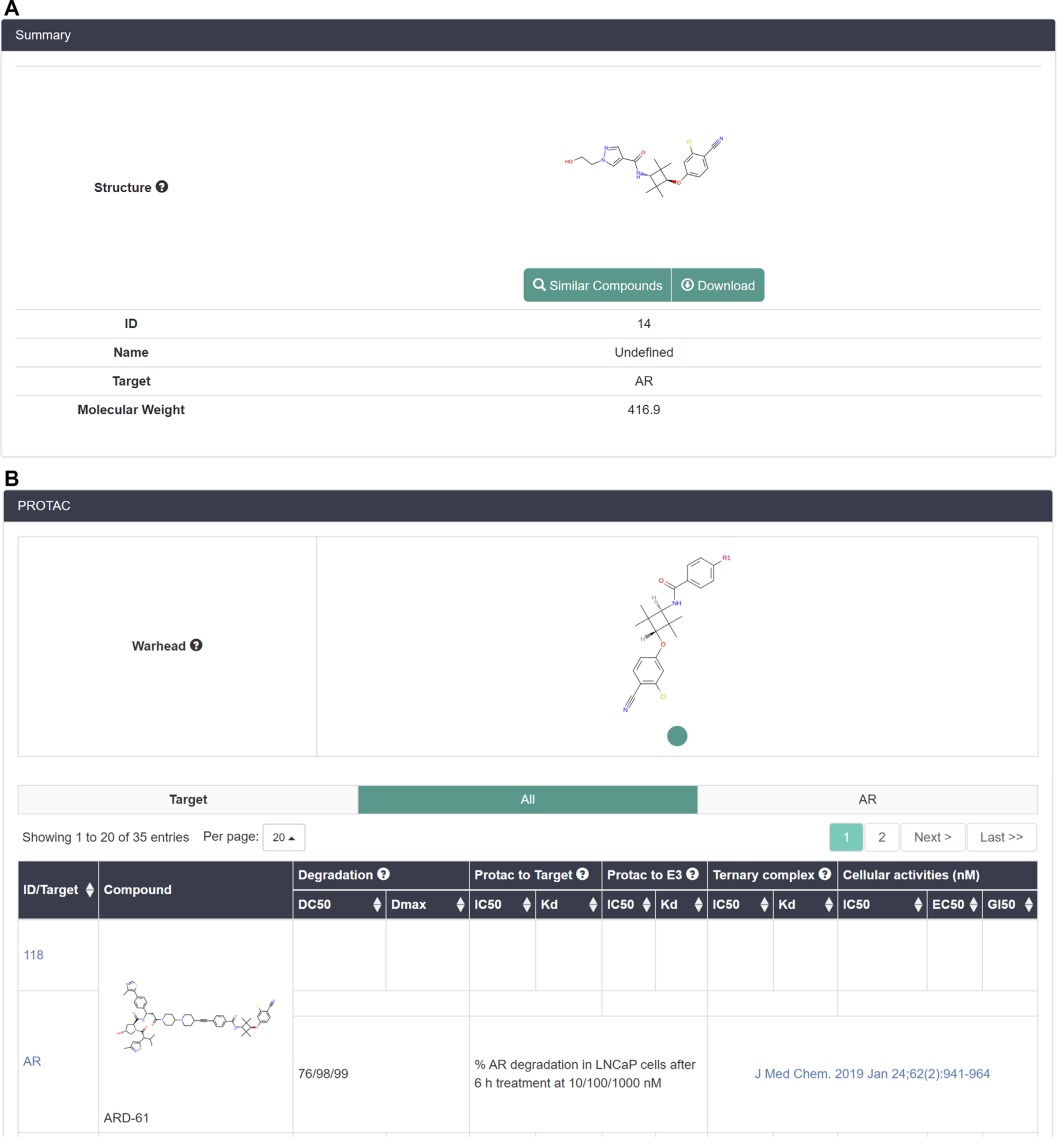
(**A**) Summary and (**B**) PROTAC tabs in the detailed information pages of warheads.

For warheads and E3 ligands, their detailed information pages are quite similar, and thus only those of warheads are described here. As shown in Figure 4A, the summary tab shows the initial structure of the warhead, compound ID, name, target, and molecular weight. Similarly, users can also look up the similar compounds in the database through the ‘Similar Compounds’ button and the similarity threshold is also ≥0.80. In addition, the MOL and SDF files of the compound are also downloadable here. For the PROTAC tab (Figure 4B), the first row displays the structure integrated into PROTACs after modification. Moreover, the PROTACs based on this warhead are summarized here to help users analyze the data easier. Users can also choose the PROTAC data for a specific target to display when this warhead targets multiple proteins. Besides, the detailed information page also contains the representation, calculated properties, activity data and external resources tabs which are not displayed in Figure 4. The representation, calculated properties, activity data tabs are similar to those of PROTACs. The external resources tab includes the links to external databases such as PubChem, ChEMBL and BindingDB.

With respect to linker, the summary tab contains the structures, compound ID, molecular weight, and buttons for similarity searching and downloading. In the PROTAC tab, similarly, the PROTACs with this linker are summarized here to help users analyze the structures. The filtering tool based on the targeted proteins is also provided here. In addition, the remaining two tabs, including representation and calculated properties, are also similar to those of PROTACs.

## CONCLUSION

Given the unique properties and potential advantages of PROTACs in the pharmaceutical sciences, data collection is necessary to address the challenge of the design of potent PROTACs. Therefore, we present a user-friendly PROTAC-DB server that enables researchers to easily query, browse and analyze the structures of PROTACs in the database. As the rapid development of PROTACs, we will constantly add new data, update the database and improve the usability of the web interface. We expect that PROTAC-DB can be served as a valuable resource and powerful tool for the rational design of PROTACs.

## References

[1] Sakamoto K.M., Kim K.B., Kumagai A., Mercurio F., Crews C.M., Deshaies R.J. Protacs: chimeric molecules that target proteins to the Skp1-Cullin-F box complex for ubiquitination and degradation. Proc. Natl. Acad. Sci. U.S.A. 2001; 98:8554–8559. 11438690 10.1073/pnas.141230798 PMC37474

[2] Schneekloth A.R., Pucheault M., Tae H.S., Crews C.M. Targeted intracellular protein degradation induced by a small molecule: en route to chemical proteomics. Bioorg. Med. Chem. Lett. 2008; 18:5904–5908. 18752944 10.1016/j.bmcl.2008.07.114 PMC3175619

[3] Schapira M., Calabrese M.F., Bullock A.N., Crews C.M. Targeted protein degradation: expanding the toolbox. Nat. Rev. Drug Discov. 2019; 18:949–963. 31666732 10.1038/s41573-019-0047-y

[4] Itoh Y., Ishikawa M., Naito M., Hashimoto Y. Protein knockdown using methyl bestatin−ligand hybrid molecules: design and synthesis of inducers of ubiquitination-mediated degradation of cellular retinoic acid-binding proteins. J. Am. Chem. Soc. 2010; 132:5820–5826. 20369832 10.1021/ja100691p

[5] Bondeson D.P., Mares A., Smith I.E.D., Ko E., Campos S., Miah A.H., Mulholland K.E., Routly N., Buckley D.L., Gustafson J.L. et al. Catalytic in vivo protein knockdown by small-molecule PROTACs. Nat. Chem. Biol. 2015; 11:611. 26075522 10.1038/nchembio.1858 PMC4629852

[6] Lai A.C., Crews C.M. Induced protein degradation: an emerging drug discovery paradigm. Nat. Rev. Drug Discov. 2017; 16:101–114. 27885283 10.1038/nrd.2016.211 PMC5684876

[7] Han X., Wang C., Qin C., Xiang W., Fernandez-Salas E., Yang C.Y., Wang M., Zhao L., Xu T., Chinnaswamy K. et al. Discovery of ARD-69 as a highly potent proteolysis targeting chimera (PROTAC) degrader of androgen receptor (AR) for the treatment of prostate cancer. J. Med. Chem. 2019; 62:941–964. 30629437 10.1021/acs.jmedchem.8b01631

[8] Li Y., Yang J., Aguilar A., McEachern D., Przybranowski S., Liu L., Yang C.Y., Wang M., Han X., Wang S. Discovery of MD-224 as a first-in-class, highly potent, and efficacious proteolysis targeting chimera murine double minute 2 degrader capable of achieving complete and durable tumor regression. J. Med. Chem. 2019; 62:448–466. 30525597 10.1021/acs.jmedchem.8b00909 PMC6545112

[9] Bai L., Zhou H., Xu R., Zhao Y., Chinnaswamy K., McEachern D., Chen J., Yang C.Y., Liu Z., Wang M. et al. A potent and selective small-molecule degrader of STAT3 achieves complete tumor regression in vivo. Cancer Cell. 2019; 36:498–511. 31715132 10.1016/j.ccell.2019.10.002 PMC6880868

[10] Winter G.E., Buckley D.L., Paulk J., Roberts J.M., Souza A., Dhe-Paganon S., Bradner J.E. Phthalimide conjugation as a strategy for in vivo target protein degradation. Science. 2015; 348:1376–1381. 25999370 10.1126/science.aab1433 PMC4937790

[11] Jiang B., Wang E.S., Donovan K.A., Liang Y., Fischer E.S., Zhang T., Gray N.S. Development of Dual and selective degraders of cyclin-dependent kinases 4 and 6. Angew. Chem. Int. Ed. Engl. 2019; 58:6321–6326. 30802347 10.1002/anie.201901336 PMC7678623

[12] Zoppi V., Hughes S.J., Maniaci C., Testa A., Gmaschitz T., Wieshofer C., Koegl M., Riching K.M., Daniels D.L., Spallarossa A. et al. Iterative design and optimization of initially inactive proteolysis targeting chimeras (PROTACs) identify VZ185 as a potent, fast, and selective von hippel-lindau (VHL) based dual degrader probe of BRD9 and BRD7. J. Med. Chem. 2019; 62:699–726. 30540463 10.1021/acs.jmedchem.8b01413 PMC6348446

[13] Raina K., Lu J., Qian Y.M., Altieri M., Gordon D., Rossi A.M.K., Wang J., Chen X., Dong H.Q., Siu K et al. PROTAC-induced BET protein degradation as a therapy for castration-resistant prostate cancer. Proc. Natl. Acad. Sci. U.S.A. 2016; 113:7124–7129. 27274052 10.1073/pnas.1521738113 PMC4932933

[14] Mullard A. Targeted degraders clear first safety hurdles. Nat. Rev. Drug Discov. 2020; 19:435. 10.1038/d41573-020-00109-w 32514100

[15] Maple H.J., Clayden N., Baron A., Stacey C., Felix R. Developing degraders: principles and perspectives on design and chemical space. Medchemcomm. 2019; 10:1755–1764. 31867093 10.1039/c9md00272c PMC6894040

[16] Edmondson S.D., Yang B., Fallan C. Proteolysis targeting chimeras (PROTACs) in ‘beyond rule-of-five’ chemical space: Recent progress and future challenges. Bioorg. Med. Chem. Lett. 2019; 29:1555–1564. 31047748 10.1016/j.bmcl.2019.04.030

[17] Chamberlain P.P., Hamann L.G. Development of targeted protein degradation therapeutics. Nat. Chem. Biol. 2019; 15:937–944. 31527835 10.1038/s41589-019-0362-y

[18] Nowak R.P., DeAngelo S.L., Buckley D., He Z., Donovan K.A., An J., Safaee N., Jedrychowski M.P., Ponthier C.M., Ishoey M. et al. Plasticity in binding confers selectivity in ligand-induced protein degradation. Nat. Chem. Biol. 2018; 14:706–714. 29892083 10.1038/s41589-018-0055-y PMC6202246

[19] Armstrong J.F., Faccenda E., Harding S.D., Pawson A.J., Southan C., Sharman J.L., Campo B., Cavanagh D.R., Alexander S.P.H., Davenport A.P. et al. The IUPHAR/BPS Guide to PHARMACOLOGY in 2020: extending immunopharmacology content and introducing the IUPHAR/MMV Guide to MALARIA PHARMACOLOGY. Nucleic. Acids. Res. 2020; 48:D1006–D1021. 31691834 10.1093/nar/gkz951 PMC7145572

[20] Kim S., Chen J., Cheng T., Gindulyte A., He J., He S., Li Q., Shoemaker B.A., Thiessen P.A., Yu B. et al. PubChem 2019 update: improved access to chemical data. Nucleic. Acids. Res. 2019; 47:D1102–D1109. 30371825 10.1093/nar/gky1033 PMC6324075

[21] Mendez D., Gaulton A., Bento A.P., Chambers J., De Veij M., Felix E., Magarinos M.P., Mosquera J.F., Mutowo P., Nowotka M. et al. ChEMBL: towards direct deposition of bioassay data. Nucleic Acids Res. 2019; 47:D930–D940. 30398643 10.1093/nar/gky1075 PMC6323927

[22] Gadd M.S., Testa A., Lucas X., Chan K.H., Chen W., Lamont D.J., Zengerle M., Ciulli A. Structural basis of PROTAC cooperative recognition for selective protein degradation. Nat. Chem. Biol. 2017; 13:514–521. 28288108 10.1038/nchembio.2329 PMC5392356

[23] Farnaby W., Koegl M., Roy M.J., Whitworth C., Diers E., Trainor N., Zollman D., Steurer S., Karolyi-Oezguer J., Riedmueller C. et al. BAF complex vulnerabilities in cancer demonstrated via structure-based PROTAC design. Nat. Chem. Biol. 2019; 15:672–680. 31178587 10.1038/s41589-019-0294-6 PMC6600871

[24] Tetko I.V., Tanchuk V.Y. Application of associative neural networks for prediction of lipophilicity in ALOGPS 2.1 program. J. Chem. Inf. Comput. Sci. 2002; 42:1136–1145. 12377001 10.1021/ci025515j

[25] Gilson M.K., Liu T., Baitaluk M., Nicola G., Hwang L., Chong J. BindingDB in 2015: a public database for medicinal chemistry, computational chemistry and systems pharmacology. Nucleic. Acids. Res. 2016; 44:D1045–D1053. 26481362 10.1093/nar/gkv1072 PMC4702793

[26] Burger M.C. ChemDoodle web components: HTML5 toolkit for chemical graphics, interfaces, and informatics. J. Cheminform. 2015; 7:35. 26185528 10.1186/s13321-015-0085-3 PMC4503857

